# Changes in Oxygen Availability during Glucose-Limited Chemostat Cultivations of *Penicillium chrysogenum* Lead to Rapid Metabolite, Flux and Productivity Responses

**DOI:** 10.3390/metabo12010045

**Published:** 2022-01-07

**Authors:** Qi Yang, Wenli Lin, Jiawei Xu, Nan Guo, Jiachen Zhao, Gaoya Wang, Yongbo Wang, Ju Chu, Guan Wang

**Affiliations:** State Key Laboratory of Bioreactor Engineering, East China University of Science and Technology (ECUST), Shanghai 200237, China; Y30190359@mail.ecust.edu.cn (Q.Y.); 10181212@mail.ecust.edu.cn (W.L.); 19000535@mail.ecust.edu.cn (J.X.); 19000406@mail.ecust.edu.cn (N.G.); y12202053@mail.ecust.edu.cn (J.Z.); y20200063@mail.ecust.edu.cn (G.W.); y30200609@mail.ecust.edu.cn (Y.W.); juchu@ecust.edu.cn (J.C.)

**Keywords:** dissolved oxygen, industrial, metabolomics, penicillin, *Penicillium chrysogenum*, scale-down

## Abstract

Bioreactor scale-up from the laboratory scale to the industrial scale has always been a pivotal step in bioprocess development. However, the transition of a bioeconomy from innovation to commercialization is often hampered by performance loss in titer, rate and yield. These are often ascribed to temporal variations of substrate and dissolved oxygen (for instance) in the environment, experienced by microorganisms at the industrial scale. Oscillations in dissolved oxygen (DO) concentration are not uncommon. Furthermore, these fluctuations can be exacerbated with poor mixing and mass transfer limitations, especially in fermentations with filamentous fungus as the microbial cell factory. In this work, the response of glucose-limited chemostat cultures of an industrial *Penicillium chrysogenum* strain to different dissolved oxygen levels was assessed under both DO shift-down (60% → 20%, 10% and 5%) and DO ramp-down (60% → 0% in 24 h) conditions. Collectively, the results revealed that the penicillin productivity decreased as the DO level dropped down below 20%, while the byproducts, e.g., 6-oxopiperidine-2-carboxylic acid (OPC) and 6-aminopenicillanic acid (6APA), accumulated. Following DO ramp-down, penicillin productivity under DO shift-up experiments returned to its maximum value in 60 h when the DO was reset to 60%. The result showed that a higher cytosolic redox status, indicated by NADH/NAD+, was observed in the presence of insufficient oxygen supply. Consistent with this, flux balance analysis indicated that the flux through the glyoxylate shunt was increased by a factor of 50 at a DO value of 5% compared to the reference control, favoring the maintenance of redox status. Interestingly, it was observed that, in comparison with the reference control, the penicillin productivity was reduced by 25% at a DO value of 5% under steady state conditions. Only a 14% reduction in penicillin productivity was observed as the DO level was ramped down to 0. Furthermore, intracellular levels of amino acids were less sensitive to DO levels at DO shift-down relative to DO ramp-down conditions; this difference could be caused by different timescales between turnover rates of amino acid pools (tens of seconds to minutes) and DO switches (hours to days at steady state and minutes to hours at ramp-down). In summary, this study showed that changes in oxygen availability can lead to rapid metabolite, flux and productivity responses, and dynamic DO perturbations could provide insight into understanding of metabolic responses in large-scale bioreactors.

## 1. Introduction

Global market values involving biotechnology have been forecasted to reach US$2–4 trillion by 2030–2040. A substantial part of the field employs microbes for production of chemicals, enzymes, foods and pharmaceuticals [1]. However, as industry has grown to rely heavily on microbes, only one in 5000–10,000 biotechnology innovations survives the long route from initial findings, through academia to product commercialization at the industrial level [2]. This valley of death in industrial biotechnology is often encountered when strains and/or processes, once optimized at the laboratory scale, are directly transferred to large-scale bioreactors for actual industrial production. This so-called scale-up effect often leads to reduced production performance in terms of titer, rate and yield (T. R. Y.), and in some cases, even batch failures.

This loss of production performance is the result of interactions between cellular metabolism and the environment in industrial-scale bioreactors. However, these are not fully accounted for during laboratory-scale process development and optimization [3,4]. Typically, most industrial fermentations employ the fed-batch mode in large-scale bioreactors with volumes reaching tens to hundreds of cubic meters. Therefore, mixing times are in the tens to hundreds of seconds due to limitations on power input and relatively long transport distances at that large scale. In contrast, cellular reaction time, which generally does not vary with the volume of the reactor, occurs on the scale of tens of seconds or even seconds [5]. When the mixing time inside the reactor is greater than or equivalent to the intracellular biochemical reaction time, large-scale fermentation processes are featured with a variety of environmental gradients, such as substrate, pH, dissolved oxygen tension (DOT) and dissolved carbon dioxide concentration [6,7].

The presence of these gradients can often lead to decreases in production capacity and the accumulation of byproducts which may be extremely detrimental to large-scale fermentation processes. To our knowledge, there have been numerous scale-down studies on the substrate concentration gradient for a wide range of industrial cell factories. These studies have provided valuable insights into cellular metabolism and suggested methods for process intensification [8,9,10,11,12,13,14]. However, unlike substrates, e.g., glucose, which can be added directly to the broth, the dissolution and consumption of oxygen involves more mass transfer and a more involved mixing process. Furthermore, the extremely low solubility of oxygen in the broth aggravates the oxygen limitation in industrial-scale bioreactors. As a consequence, the dissolved oxygen gradient in the reactor is likely to occur with the presence of substrate gradient. Oxygen is essential for all aerobic microbes because it is involved in the oxidative phosphorylation process as the final electron acceptor in the electron transport chain. Under hypoxic conditions, oxygen deprivation leads to metabolic shift into anaerobic fermentation pathways. This hypoxic shift is mostly detrimental to cellular growth and product formation, but can be beneficial in some cases. A few studies reported the effects of the availability of dissolved oxygen levels on overall productivity and quality. For example, fluctuations in dissolved oxygen are very harmful to production processes using *E. coli*, where exposure to low dissolved oxygen for only 13 s is sufficient to induce gene expression in the anaerobic pathway and produce a large number of byproducts [15]. DOT fluctuations can also affect the properties of products. Serrato et al. reported that oscillating DOT affected the N-glycosylation pattern of monoclonal antibodies produced in hybridoma cultures, thus affecting their effector functions [16]. Meanwhile, Trujillo-Roldánet et al. found that the magnitude of DOT fluctuations had an important effect on the molecular weight of alginate produced by *Azotobacter vinelandii*. Therefore, alginates of different molecular weights can be obtained by controlling different levels of DOT [17]. However, in some cases, it has been demonstrated that DOT fluctuations can be beneficial for growth and product synthesis. Diano et al. [18] reported a study on the production of polyols by continuous fermentation of *Aspergillus niger* under oxygen-limited conditions. The results showed that sugar polyols (xylitol, erythritol and arabitol) formed in the cell could account for up to 22% of the carbon source consumed. Polyol production is mainly used to regulate the intracellular metabolism of *Aspergillus niger*, which are synthesized as energy and storage substances when the flux in the intracellular pentose phosphate (PP) pathway exceeds that required for biomass synthesis. In 2012, Pedersen et al. investigated the effects of dissolved oxygen levels on the production of glucoamylase by *Aspergillus niger* during fed-batch fermentation, and found that proper oxygen limitation could increase the yield of glucoamylase. The yield based on a carbon source increased because the byproducts (such as polyols) could be reconsumed and improved the utilization of the carbon source [19]. In the actual production process, the dissolved oxygen level needed to be controlled and kept neither too high nor too low in order to keep it from affecting the overall production performance of microbial cell factories. In 2018, Lu et al. investigated the metabolism of glucoamylase production in *Aspergillus niger* during hypoxia, based on the latest GEM (Genome-scale metabolic model). They used a multi-omics analysis approach, including metabolomics, flux omics, and transcriptomics [20]. The results suggested that a possible reason for the rise in glucoamylase production was the elevation of enzyme precursor amino acids under hypoxic conditions. Additionally, glyoxylate shunt gene expression was found to be activated to reduce the production of NADH in the TCA cycle and to facilitate the maintenance of cellular redox homeostasis under hypoxic conditions.

*Penicillium chrysogenum* has been exploited as the main cell factory for industrial penicillin production. During the biosynthesis of penicillin, oxygen is not only consumed as the electron acceptor for provision of ATP (oxidative phosphorylation process), required for cell growth, but also serves as a direct precursor in the second step (from ACV to IPN) of the penicillin biosynthetic pathway. Therefore, the supply of oxygen plays a direct role in both cell growth and penicillin production [21]. Typically, aerobic fermentation for antibiotic production (e.g., penicillin, cephalosporins, and streptomycin) is usually carried out in large stirred fermenters with volumes up to 300 m^3^ [22]. These filamentous fungal fermentations feature increased viscosity of the broth and non-Newtonian fluid properties. In this highly viscous environment, oxygen transfer is limited, even in the vicinity of the stirring paddle, leading to the appearance of dissolved oxygen gradients in the fermenter. In previous studies, this localized hypoxic state, observed in large-scale reactors, was difficult to reproduce in laboratory-scale reactors, leading to blind spots in research [23,24]. To the best of our knowledge, although a few studies have reported on the effects of dissolved oxygen fluctuation on cellular metabolism and penicillin production, they produced only limited observations regarding cellular regulation mechanisms. For example, Henriksen et al. [25] studied the influence of dissolved oxygen concentration on penicillin biosynthesis in steady-state continuous cultures of a high-yielding strain of *Penicillium chrysogenum* operated at a dilution rate of 0.05 $h^{-1}$. Their results showed that, at dissolved oxygen concentrations above 0.06–0.08 mM, a constant specific penicillin productivity of around 22 µmol/gDW/h was maintained. At lower oxygen concentrations, the specific penicillin productivity decreased, and a value of 17 µmol/gDW/h was obtained when the dissolved oxygen concentration was 0.042 mM. However, penicillin productivity was instantly recovered to its maximum value when the dissolved oxygen concentration was reset to above 0.08 mM. The specific excretion rates of byproducts associated with the penicillin production increased with decreasing dissolved oxygen concentration. However, different dissolved oxygen experiments were carried out in the same batch for a short time, which may have interfered with results between each chance in the dissolved oxygen level. Furthermore, due to the limited experimental conditions, they did not explain the reasons for the decline of penicillin production using a systems approach in detail. Vardar and Lilly [26] studied the effects of periodic fluctuations in dissolved oxygen levels on penicillin production and the respiratory metabolism of the cell using *Penicillium chrysogenum*. The findings revealed that below 30% saturated dissolved oxygen, the rate of penicillin synthesis rapidly decreased, and below 10% saturated dissolved oxygen, there was no penicillin synthesis. The effect was irreversible. However, there were certain irrationalities in this experimental design, such as the use of pressure cycling fluctuations to achieve dissolved oxygen fluctuations. Under the same pressure and temperature environment, CO_2_ solubility in the fermentation broth (38 mol CO_2_/m^3^/bar (CO_2_)) was 30 times higher than oxygen solubility (1.25 mol O_2_/m^3^/bar (O_2_)), which may have directly led to a more substantial cyclic fluctuation of CO_2_ concentration in the fermenter. In addition, their study used the fed-batch fermentation mode, which was accompanied by changes in biomass concentration, product concentration and even limiting factors.

In this study, the response of glucose-limited chemostat cultures of an industrial strain of *Penicillium chrysogenum* to different dissolved oxygen levels was assessed. To achieve this, a high-producing industrial strain was cultivated at a growth rate of 0.05 $h^{-1}$ in glucose-limited chemostat cultures, both under different steady state DO levels and DO ramp-down conditions. Furthermore, to obtain a holistic view of DO dynamics on the relation between central metabolism, amino acid biosynthesis and penicillin production in *Penicillium chrysogenum*, a systems approach using fluxomics (stoichiometry) and metabolomics for DO shift-down and DO ramp-down conditions was carried out—for the first time—in order to study the biological effects of changing DO levels for this microorganism.

## 2. Results

### 2.1. DO-Shift Experiments

#### 2.1.1. General Observations

**Figure 1:**
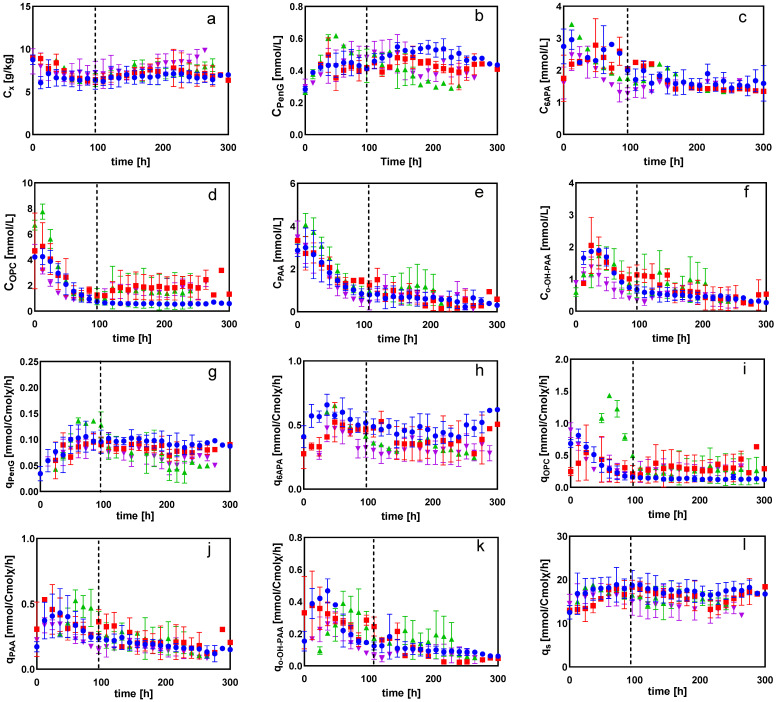
Measured concentrations of (**a**) biomass, (**b**) Penicillin G (PenG), (**c**) 6-aminopenicillanic acid (6APA), (**d**) 6-oxopiperidine-2-carboxylic acid (OPC), (**e**) phenylacetic acid (PAA), (**f**) οrtho-hydroxyphenyl acetic acid (ο-OH-PAA), (**g**) the biomass-specific penicillin production rate ($q_{\mathrm{PenG}}$), (**h**) the biomass-specific 6-aminopenicillanic acid production rate ($q_{6\mathrm{APA}}$), (**i**) the biomass-specific 6-oxopiperidine-2-carboxylic acid production rate ($q_{\mathrm{OPC}}$), (**j**) the biomass-specific phenylacetic acid consumption rate ($q_{\mathrm{PAA}}$), (**k**) the biomass-specific ο-OH-PAA production rate ($q_{ο-\mathrm{OH}-\mathrm{PAA}}$) and (**l**) the biomass-specific substrate consumption rate ($q_{s}$) at DO-shift experiments. The dashed line represents the start of DO-shift experiment following 100 h chemostat cultivation at DO of 60%. Time 0 represents the start of chemostat cultivation. Different symbols signify different DO-shift experiments with (●) 60%, (■) 20%, (▲) 10%, (▼) 5%.

**Figure 2:**
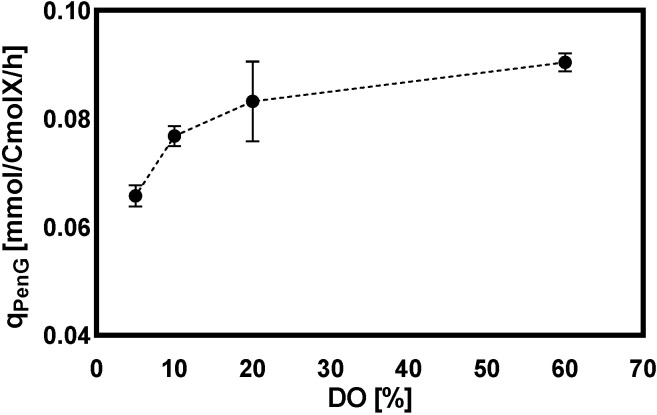
The biomass-specific PenG production rate ($q_{\mathrm{PenG}}$) at DO-shift experiments, and the data at different DO levels are the mean ± standard deviation of at least three replicate experiments.

After the depletion of glucose in the batch phase, chemostat cultivation was initiated. The control group, with 60% dissolved oxygen, was maintained throughout the cultivation process, while the DO-shift experiments were carried out after 100 h of chemostat cultivation at the DO of 60%. The concentrations of biomass, penicillin G (PenG), phenylacetic acid (PAA), OPC, o-OH-PAA, 6-APA and their biomass-specific consumption/production rates are shown in Figure 1 for the chemostat process with PAA as the precursor and continuous supplementation with other media components for the synthesis of PenG. It was evident from these chronological data that all the chemostat cultivations were very reproducible. The residual concentration of PAA in all batches was at the level of 1–4 mM and was neither toxic to the cells nor restrictive to the synthesis of PenG [27]. As seen in Figure 1**,** the biomass-specific PenG production rate reached its highest value after the initiation of the chemostat culture of *Penicillium chrysogenum* at 70–90 h, which was associated with the induction of expression of the penicillin pathway synthesis gene [14]. Throughout the cultivation, the $q_{\mathrm{PenG}}$ values were 25%, 14% and 7% lower at 5%, 10% and 20% than at 60% dissolved oxygen conditions, respectively. This indicated that the dissolved oxygen concentration affected the production rate of penicillin—the lower the dissolved oxygen, the lower the $q_{\mathrm{PenG}}$. One previous study reported the effects of periodic fluctuations in dissolved oxygen levels on PenG biosynthesis with *Penicillium chrysogenum* and the respiratory metabolism of the cell. The results showed that, below 30% saturated dissolved oxygen, the $q_{\mathrm{PenG}}$ decreased rapidly [26]. However, our result showed that $q_{\mathrm{PenG}}$ decreased significantly (*p* < 0.0001) only when the dissolved oxygen concentration was lower than 10% (Figure 2). This difference may be ascribed to different strains used, as well as different cultivation conditions. Additionally, the biomass curve showed that, at 60% dissolved oxygen concentration, the biomass was about 6 g/kg, while at 5% dissolved oxygen, the biomass reached about 8 g/kg (Figure 1). The rise of biomass concentrations at low DO levels may be associated with the loss of capacity to produce PenG, at which point more carbon and energy can be directed towards biomass growth. Consistently, van Gulik et al. found that, in carbon-limited chemostat cultivations of a high-yielding *Penicillium chrysogenum* strain, the loss of penicillin production was observed and was accompanied by the gradual increase of biomass concentration. A possible reason for this could be the gradual replacement of high-yielding strains by non-yielding and low-yielding strains [28]. Additionally, the calculated biomass yield ($Y_{X/S}$) showed an inverse relationship with penicillin production capacity (Table 1). At the same time, the concentration of the three main byproducts (6-APA, o-OH-PAA, and OPC) and their specific production/consumption rates increased with decreasing dissolved oxygen concentration, the most obvious of which was the increase of OPC by 38% at 5% dissolved oxygen, compared to DO at 60%. This was reasonable, given that the loss of PenG production capacity gives rise to the accumulation of one of the amino acid precursors, α-aminoadipic acid, in large quantities, under a hypoxic environment, thus cyclizing itself to form OPC and excreting it from the cell. Likewise, the concentration of the precursor PAA increased due to the decrease of penicillin production capacity under hypoxia, which was consistent with the results of our previous study [29].

Table 1 shows the specific rates and relevant yields for glucose-limited chemostat cultures of *Penicillium chrysogenum* at DO-shift conditions. The biomass-specific production rates of penicillin (and major byproducts such as 6-APA and OPC) differed with decreasing dissolved oxygen concentrations. When the dissolved oxygen concentration was shifted from 60% to 20%, 10% and then 5%, the $q_{\mathrm{PenG}}$ was reduced by 10%, 15%, and 25%, respectively. Strikingly, it was also noted that the specific oxygen consumption rate ($q_{O_{2}}$) and the specific carbon dioxide production rate ($q_{{\mathrm{CO}}_{2}}$) were reduced by 16% when the dissolved oxygen concentration was shifted from 60% to 5%, and the substrate consumption rate ($q_{s}$) was reduced by 18%.

#### 2.1.2. Intracellular and Extracellular Metabolites

**Figure 3:**
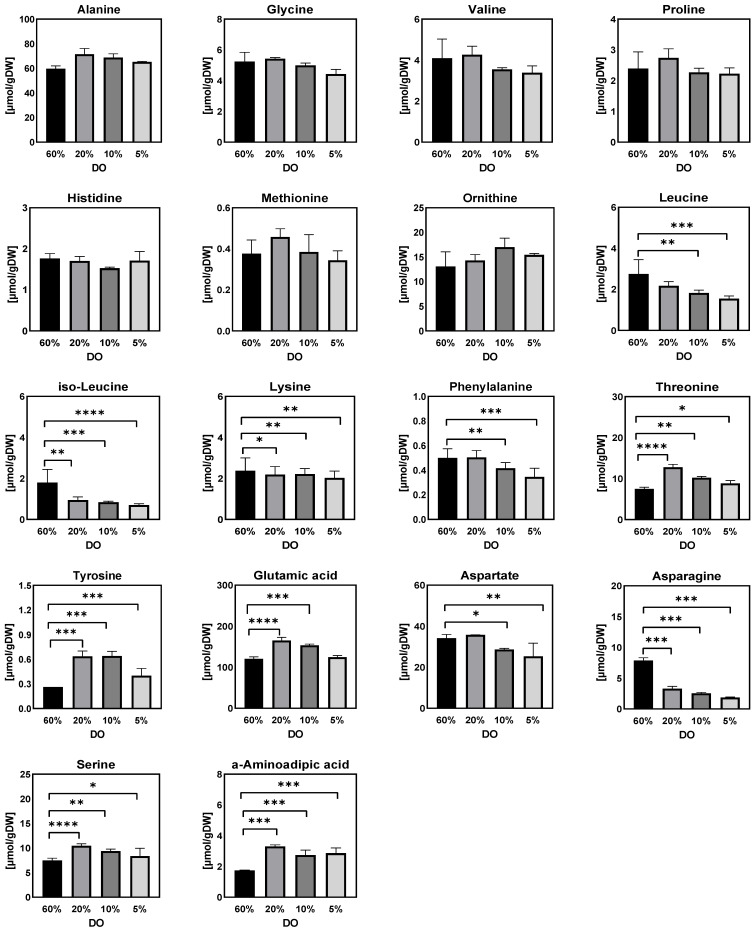
Intracellular amounts of amino acids in *Penicillium chrysogenum* Wisconsin 54-1255 under steady-state dissolved oxygen (60%, 20%, 10% and 5%) conditions. Samples were taken at 24 h intervals and data were collected from three replicate experiments; Two-tailed Student’s *t*-tests ((*) *p* < 0.05, (**) *p* < 0.01, (***) *p* < 0.001 and (****) *p* < 0.0001) were performed using GraphPad Prism 8.

**Figure 4:**
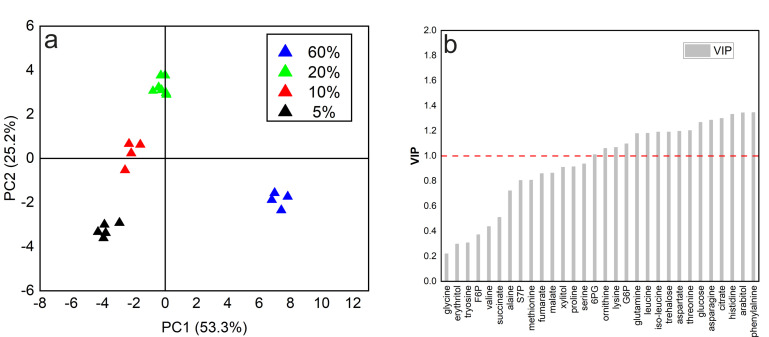
Analysis of metabolomics data under different steady-state dissolved oxygen concentrations. (**a**) Principal component analysis (PCA)-derived score plot of the first two principal components and (**b**) VIP score for 30 intracellular metabolites calculated with the PLS model.

**Figure 5:**
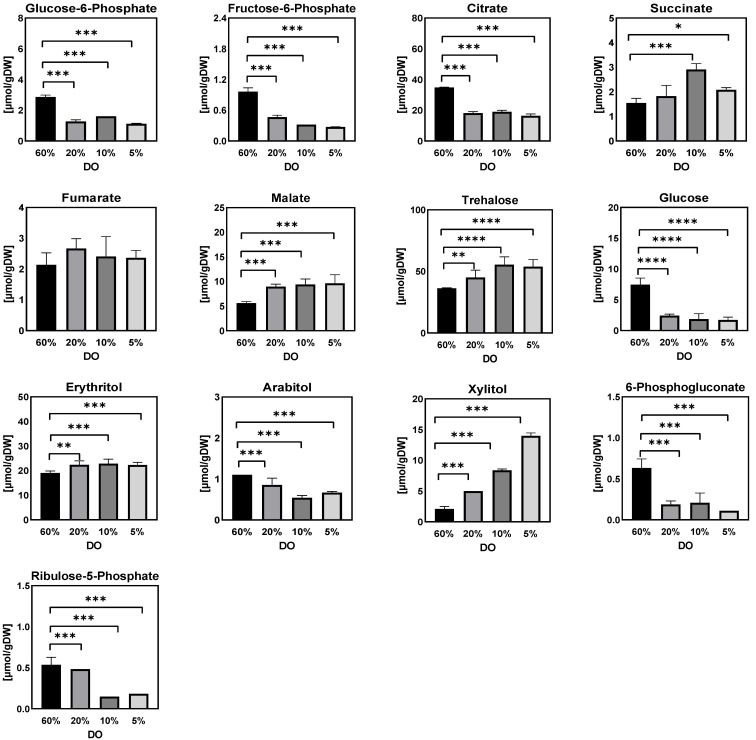
Intracellular amounts of sugar phosphates, organic acids, and sugar alcohols in *Penicillium chrysogenum* Wisconsin 54-1255 under steady-state dissolved oxygen (60%, 20%, 10%, and 5%). Samples were collected at 24 h intervals and data were collected from three replicate experiments; Two-tailed Student’s *t*-tests ((*) *p* < 0.05, (**) *p* < 0.01, (***) *p* < 0.001 and (****) *p* < 0.0001) were performed using GraphPad Prism 8.

**Figure 6:**
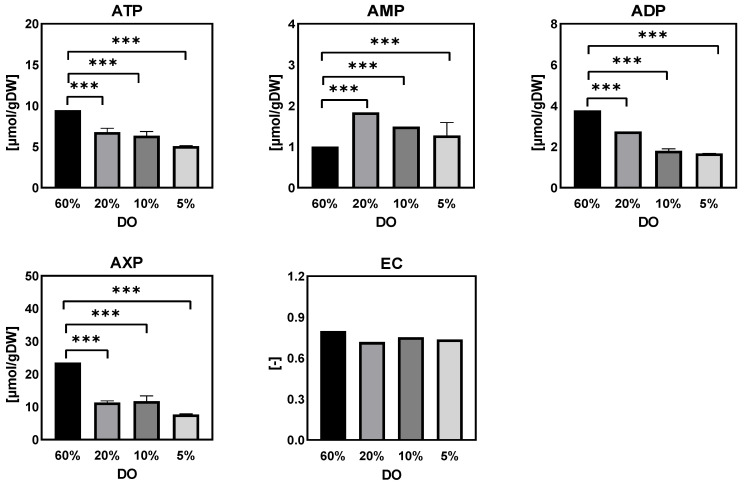
Intracellular amounts of adenine nucleotides and the energy charge in *Penicillium chrysogenum* Wisconsin 54-1255 under steady-state dissolved oxygen (60%, 20%, 10%, and 5%) conditions. Samples were collected at 24 h intervals and data were collected from three replicate experiments; Two-tailed Student’s *t*-tests ((***) *p* < 0.001) were performed using GraphPad Prism 8.

**Figure 7:**
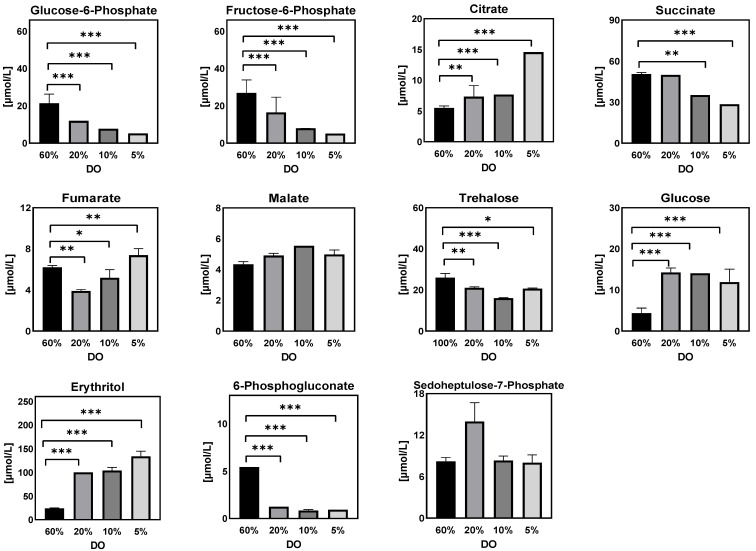
Extracellular concentrations of sugar phosphates, organic acids, and sugar alcohols in *Penicillium chrysogenum* Wisconsin 54-1255 under steady-state dissolved oxygen (60%, 20%, 10%, and 5%) conditions. Samples were collected at 24 h intervals and data were collected from three replicate experiments; Two-tailed Student’s *t*-tests ((*) *p* < 0.05, (**) *p* < 0.01 and (***) *p* < 0.001) were performed using GraphPad Prism 8.

**Figure 8:**
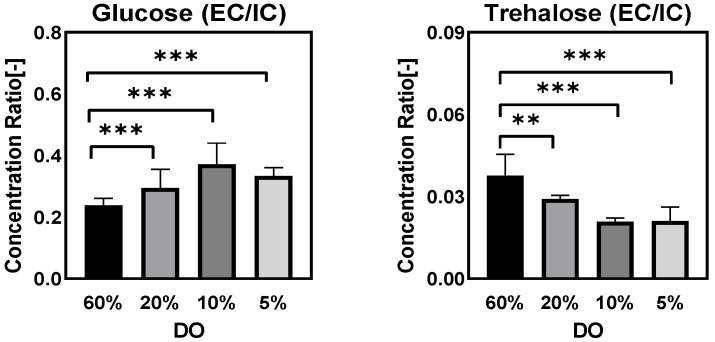
The ratio of extra/intracellular (EC/IC) trehalose and glucose concentrations (assuming a cellular volume of 2.5 mL/gDW) in *Penicillium chrysogenum* Wisconsin 54-1255 under steady-state dissolved oxygen (60%, 20%, 10%, and 5%) conditions. Samples were collected at 24 h intervals and data were collected from three replicate experiments. Two-tailed Student’s *t*-tests ((**) *p* < 0.01 and (***) *p* < 0.001) were performed using GraphPad Prism 8.

Figure 3, Figure 4, Figure 5, Figure 6, Figure 7 and Figure 8 show the intracellular amounts of amino acids, sugar phosphates, organic acids, sugar alcohols and adenine nucleotides, as well as extracellular concentrations of sugar phosphates, organic acids, and sugar alcohols under different steady-state dissolved oxygen conditions. The results showed that the intra- and extracellular metabolites displayed different kinetic responses under the four different steady-state dissolved oxygen conditions. Under low dissolved oxygen conditions, the most obvious differences at different concentrations were the intracellular and extracellular levels of sugar phosphates, sugar alcohols, adenine nucleotides and some organic acids (Figure 5, Figure 6, Figure 7 and Figure 8). By contrast, only half the intracellular amounts of amino acid did not show significant changes with different dissolved oxygen levels (Figure 3), partly because the amino acid pool was relatively large and has a long turnover time.

Compared to a similar low dissolved oxygen steady-state cultivation in *Aspergillus niger* under industrial enzyme production conditions [20], similarities were observed in the changes of intra- and extracellular metabolites after the cells experienced low dissolved oxygen conditions, as follows: (i) decrease in intracellular amounts of glycolytic intermediates such as G6P and F6P; (ii) moderate decrease of metabolites in the TCA cycle and (iii) significant increase in extracellular concentrations of sugar alcohols. Nonetheless, differences in the responses of the different microorganisms to low dissolved oxygen levels were reflected in the increase and decrease of intracellular sugar alcohols in *Aspergillus niger* and *Penicillium chrysogenum*, respectively. As regards different categories of measured metabolites, their responses to different dissolved oxygen levels were also different, as follows:

(a) Amino acids

Under low dissolved oxygen conditions, the dynamics in the size of the intracellular amino acid pool showed two different trends, with leucine, isoleucine, aspartate, glycine, and phenylalanine decreasing significantly while serine, threonine, glutamic acid, and tyrosine significantly increased. In addition, the principal component analysis (PCA) showed that all samples could be divided into four groups in accordance with four different dissolved oxygen levels (Figure 4a). On the other hand, the relationship between metabolite pool size changes and the specific oxygen uptake rate ($q_{O_{2}}$) was analyzed using partial least squares (PLS) method. The results showed that 16 metabolites of a total of 30 metabolites measured had a VIP score above 1, indicating that most intracellular metabolite concentration changes were sensitive to external oxygen perturbations (Figure 4b).

(b) Sugar phosphates, organic acids, and sugar alcohols

Glucose-6-phosphate, fructose-6-phosphate, ribulose-5-phosphate, and 6-phosphogluconate showed a decreasing trend at low DO levels, potentially favoring flux through the EMP pathway and relieving energy demands for cell metabolism in the presence of higher redox level status at low DO levels. This was consistent with the results of *Aspergillus niger* under low oxygen conditions [20]. In addition, the intracellular amounts of citric acid, malic acid and succinate were higher while there were marginal amounts of 2-oxoglutarate (below detection limits) under low DO conditions, implying a more pronounced increase in the flux via glyoxylate cycle under low dissolved oxygen conditions. This will be further validated by flux balance analysis.

Furthermore, intracellular amounts of storage sugars (erythritol, arabinitol, mannitol and xylitol) were significantly increased with decreasing DO concentration, while the intracellular glucose level showed an opposite trend (Figure 5). Numerous studies have reported the important roles of these sugar alcohols in a wide range of microorganisms [30,31,32,33]. For example, in *Penicillium chrysogenum*, Wang et al. showed that the periodic synthesis/degradation of trehalose in a perturbed environment required the consumption of additional ATP, but higher intracellular trehalose level facilitated the rapid adaptation of the cell to the extracellular fluctuating environment and maintained the stability of the intracellular metabolism [29]. In this study, these sugar alcohols were important sinks of carbon/energy sources and aided in reducing power to counteract the hypoxic environment. However, it should be noted that the physiological role of the pathways leading to these polyols are fundamentally metabolic, i.e., more complex than simply the consequence of increased concentrations of sugars.

(c) Purine nucleotide and energy charge

It was apparent that the intracellular amounts of purine nucleotides (ATP, ADP and AMP) significantly decreased as the DO level decreased from 60% to 20%, 10% and 5% (Figure 6). As a result, the total amount of AXP was almost halved under these low DO levels as compared to the reference control. However, the energy charge was essentially unchanged at different steady-state DO levels. In *Penicillium chrysogenum*, van Gulik et al. reported that, for the production of 1 mol penicillin G, 73 mol ATP was required [28]. Hence, penicillin production is a high ATP-consuming process and requires a sufficient supply of ATP and a high energy charge. In this study, under low dissolved oxygen levels, the low energy status partially contributed to the decreased penicillin productivity.

(d) Extracellular sugar phosphates, organic acids, and sugar alcohols

In general, the change of extracellular metabolites followed the dynamics of their intracellular counterparts (Figure 7). Under low dissolved oxygen conditions, the extracellular concentrations of sugar phosphate varied greatly, with G6P, 6PG, succinic acid, fumaric acid, and trehalose showing monotonous increase while glucose and erythritol showed monotonous decrease. Remarkably, the extracellular glucose concentration was increased fourfold when dissolved oxygen was reduced from 60% to 5%.

Figure 8 gives the ratio of extra/intracellular (EC/IC) concentrations regarding glucose and trehalose. The results indicated that glucose was transported into the cells less, which was consistent with the decrease of the biomass-specific glucose consumption rate observed as the DO level decreased (Table 1). However, trehalose exhibited an opposite trend, both intracellularly and extracellularly. When the DO level decreased, the EC/IC concentrations of trehalose indicated that *Penicillium chrysogenum* cells were inclined to keep trehalose inside the cell rather than excreting it into the environment.

##### Intracellular Metabolic Flux Distribution

Metabolic fluxes and metabolite concentrations, measured by metabolomics, complement each other for quantitative understanding of cellular metabolism [34]. Genome-scale models play an important role in quantitatively predicting cellular metabolism and further exploring the improved properties of production strains through metabolic engineering [35].

**Figure 9:**
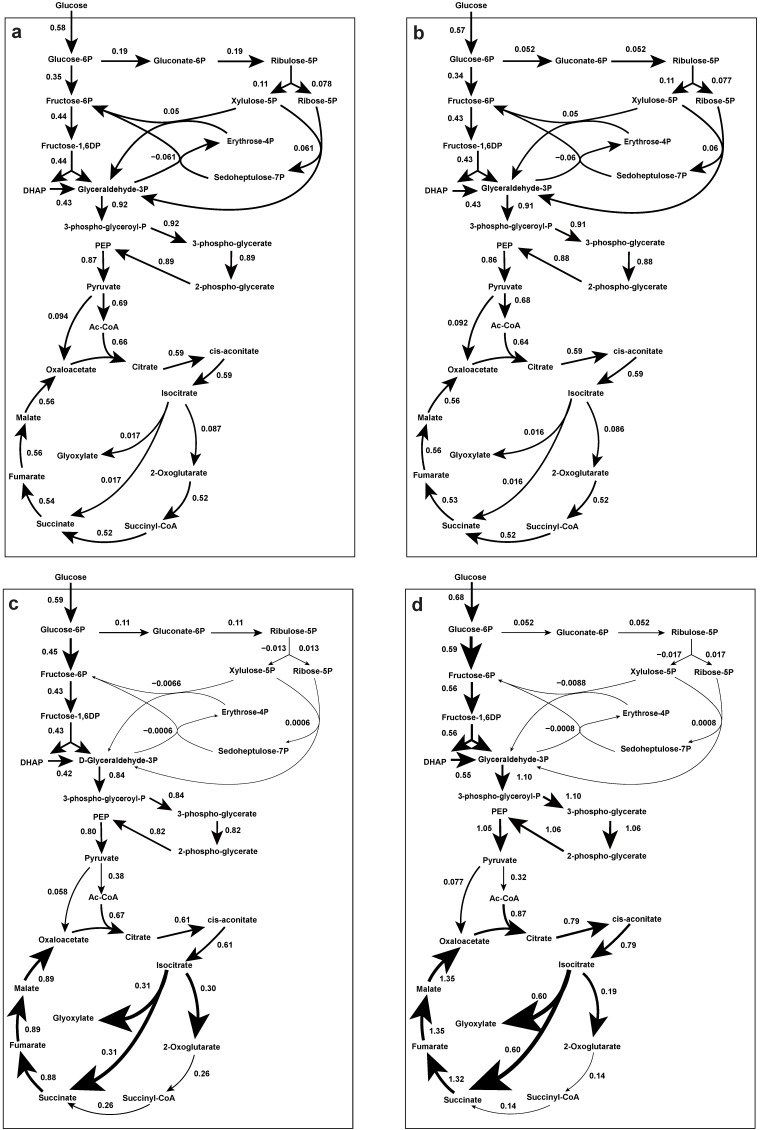
Fluxes (mmol/gDW/h) of *P. chrysogenum* Wisconsin 54-1255 through the central metabolic pathway calculated with the genome-scale metabolic model (IAL1006) using the RAVEN toolbox. (**a**) 60% dissolved oxygen, (**b**) 20% dissolved oxygen, (**c**) 10% dissolved oxygen and (**d**) 5% dissolved oxygen. The specific rates calculated in Table 1 are used as model inputs, with maximization of cell growth as the objective function. The direction of the arrow indicates the direction of the flux and the thickness of the arrow indicates the magnitude of the flux.

In this study, the previously established genome-scale metabolic model of *Penicillium chrysogenum* Wisconsin 54-1255 was used to estimate the flux distribution [36]. Figure 9 shows the metabolic fluxes at 60%, 20%, 10% and 5% dissolved oxygen conditions. We observed that the flux from isocitrate to glyoxylate in the TCA cycle at 5% DO was 30 times higher than at 60% DO, while the flux from α-ketoglutarate to succinyl coenzyme A significantly decreased. This indicated that oxygen limitation downregulated part of the TCA cycle and that the glyoxylate branch was activated. This was consistent with the results of *E. coli* expressing green recombinant protein under DOT fluctuation, which caused upregulation of ketoglutarate synthase and isocitrate lyase expression, resulting in a flux shift to the glyoxylate cycle to reduce intracellular NADH production [37]. Therefore, the observed rise in intracellular flux through the glyoxylate shunt may have been a cellular effort to resist the unfavorable hypoxic environment.

In contrast, the flux branching off from G6P to the pentose phosphate (PP) pathway was about two times higher under 60% dissolved oxygen conditions than under 5%. This result was consistent with the approximately 2-fold higher PenG production rate under 60% as compared to 5% dissolved oxygen conditions. This was reasonable, given that high NADPH requirements have been reported for higher PenG production [36].

### 2.2. DO-Ramp Experiments

#### 2.2.1. General Observations

**Figure 10:**
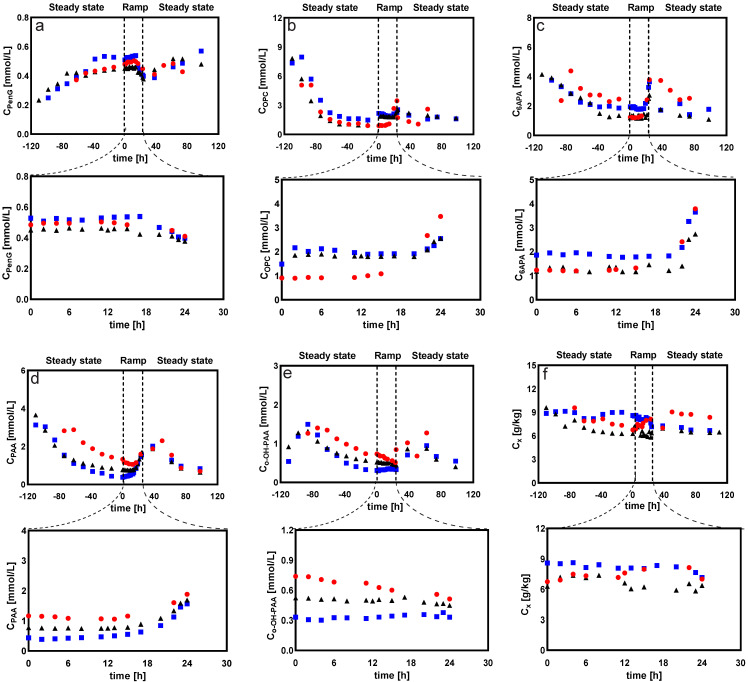

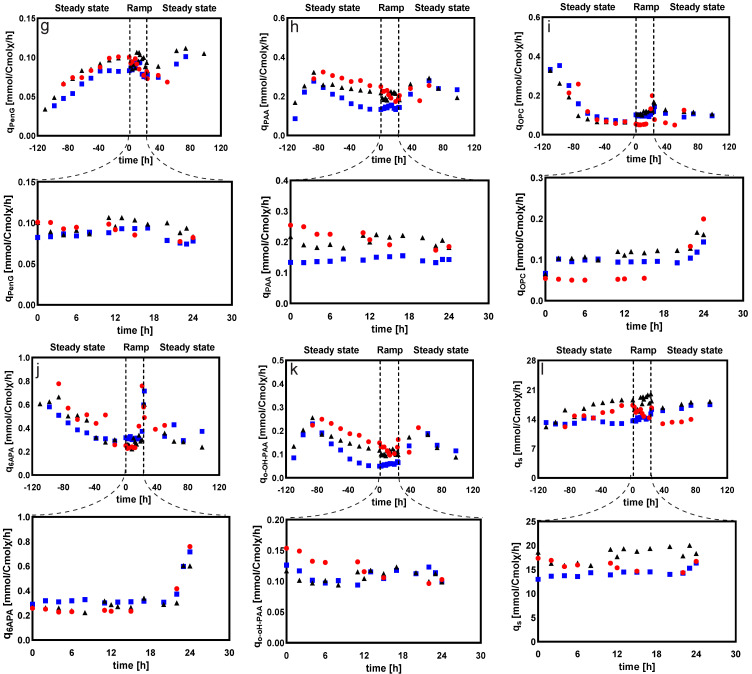
Measured concentrations of (**a**) Penicillin G (PenG), (**b**) 6-oxopiperidine-2-carboxylic acid (OPC), (**c**) 6-aminopenicillanic acid (6APA), (**d**) phenylacetic acid (PAA), (**e**) οrtho-hydroxyphenyl acetic acid (ο-OH-PAA), (**f**) biomass ($C_{X}$), (**g**) the biomass-specific penicillin production rate ($q_{\mathrm{PenG}}$), (**h**) the biomass-specific phenylacetic acid consumption rate ($q_{\mathrm{PAA}}$), (**i**) the biomass-specific 6-oxopiperidine-2-carboxylic acid production rate ($q_{\mathrm{OPC}}$), (**j**) the biomass-specific 6-aminopenicillanic acid production rate ($q_{6\mathrm{APA}}$), (**k**) the biomass-specific ο-OH-PAA production rate ($q_{ο-\mathrm{OH}-\mathrm{PAA}}$) and (**l**) the biomass-specific substrate consumption rate ($q_{s}$) during DO-ramp experiments with *Penicillium chrysogenum* Wisconsin 54-1255. DO: steady state phases (−120–0 h, 60%), ramp-down phases (0–24 h, linearly decreased from 60% to 0%), and DO recovery phases (24–120 h, shifted up to 60%). Three legends represent three replicate experiments.

To further investigate the effects of changing DO on the cellular regulation mechanism in *Penicillium chrysogenum*, DO ramp experiments, based on glucose-limited chemostats, were carried out to generate a large metabolite dataset across a wide range of DO conditions. According to the DO control strategy, the chemostat cultivation process was divided into three phases. The DO was maintained at 60% at the first stage (−120 h–0 h, steady state), then linearly decreased from 60% to 0% over the following 24 h (0–24 h, ramp down). Finally, the DO was shifted up to 60% and maintained for observation time (24–100 h, shift up). Figure 10 shows the dynamics of product and byproduct concentrations, as well as the biomass-specific production/consumption rates. The results showed that PenG first increased and then leveled off in the first phase. It began to decrease as the DO dropped to 10%, and the $q_{\mathrm{PenG}}$ decreased by 15% at the end of phase II. During the DO recovery phase, the PenG concentration and $q_{\mathrm{PenG}}$ gradually returned to the values observed at the onset of phase II. This indicated that *Penicillium chrysogenum* Wisconsin 54-1255 was capable of restoring penicillin productivity after undergoing a short period of low DO levels. These results were in agreement with the results of our previous study [26] and further proved that *Penicillium chrysogenum* is robust against low DO levels.

During the linear decrease of dissolved oxygen levels, byproducts such as 6APA and OPC significantly accumulated (respectively, these were about 50% and 25% higher at the end of phase II than at the onset). This was reasonable; as penicillin productivity was impaired in response to low DO levels, one of the three precursor amino acids, αAAA, could be recycled to form OPC. Additionally, 6APA was excreted outside the cell without further incorporation into PenG [38]. In addition, a gradual increase in the extracellular PAA was observed, indicating that, as penicillin productivity decreased in response to the low DO level, more precursor PAA was accumulated. This was consistent with the results of DO-shift experiments in this study. However, the $q_{\mathrm{PenG}}$ decreased by 15% when the DO level was linearly reduced to 0% over 24 h, while the $q_{\mathrm{PenG}}$ was 25% lower when the DO was shifted to 5% compared to its levels when DO was 60%. This implied that, aside from DO levels, in *Penicillium chrysogenum,* the extent of loss of penicillin productivity in response to low DO perturbation also depends on exposure duration. Consistent with this, it was observed that the byproducts associated with penicillin production accumulated in higher amounts under prolonged DO-shift than under DO-ramp experiments (Figure 1 and Figure 10).

**Figure 11:**
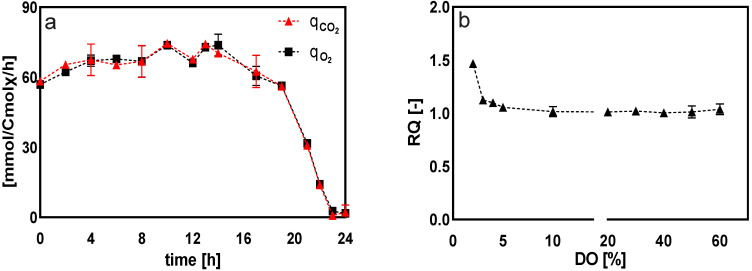
(**a**) Changes in the respiratory intensity of the cell at DO-ramp experiments. The dissolved oxygen was linearly decreased from 60% to 0% in 24 h. Time 0 represents the startup of DO ramp-down; (**b**) Respiratory quotient of *Penicillium chrysogenum* Wisconsin 54-1255 at different dissolved oxygen levels calculated from DO shift and ramp experiments.

Figure 11 compares the respiratory metabolism of the cell at different dissolved oxygen levels. The results indicated that *Penicillium chrysogenum* had a very high affinity for dissolved oxygen, which was macroscopically demonstrated by the fact that its respiratory intensity did not decrease with the decrease of dissolved oxygen concentration. Instead, a small increase, indicated by the $q_{O_{2}}\;$and $q_{{\mathrm{CO}}_{2}}$, was observed before DO was decreased to 10%. However, when the DO was less than 5%, the $q_{O_{2}}\;$and $q_{{\mathrm{CO}}_{2}}$ decreased almost linearly with the decreasing DO level (Figure 11a). Thus, we concluded that the strain was highly adaptable to different dissolved oxygen levels and that its respiratory intensity did not fluctuate drastically with decreasing dissolved oxygen levels. As seen in Figure 11b, the respiratory quotient of the cell sharply increased with decreasing dissolved oxygen levels (less than 5%). This increase could have been caused by the accumulation of reduced metabolites (in the form of polysaccharides such as trehalose, mannitol, and erythritol)—which was observed in DO shift experiments (Figure 5). In *Penicillium chrysogenum*, previous studies have shown that these sugar alcohols can fulfill diverse physiological roles, serving as an overflow reservoir for reducing power as well as stress tolerance [32].

#### 2.2.2. Intracellular and Extracellular Metabolites

##### Amino Acids

**Figure 12:**
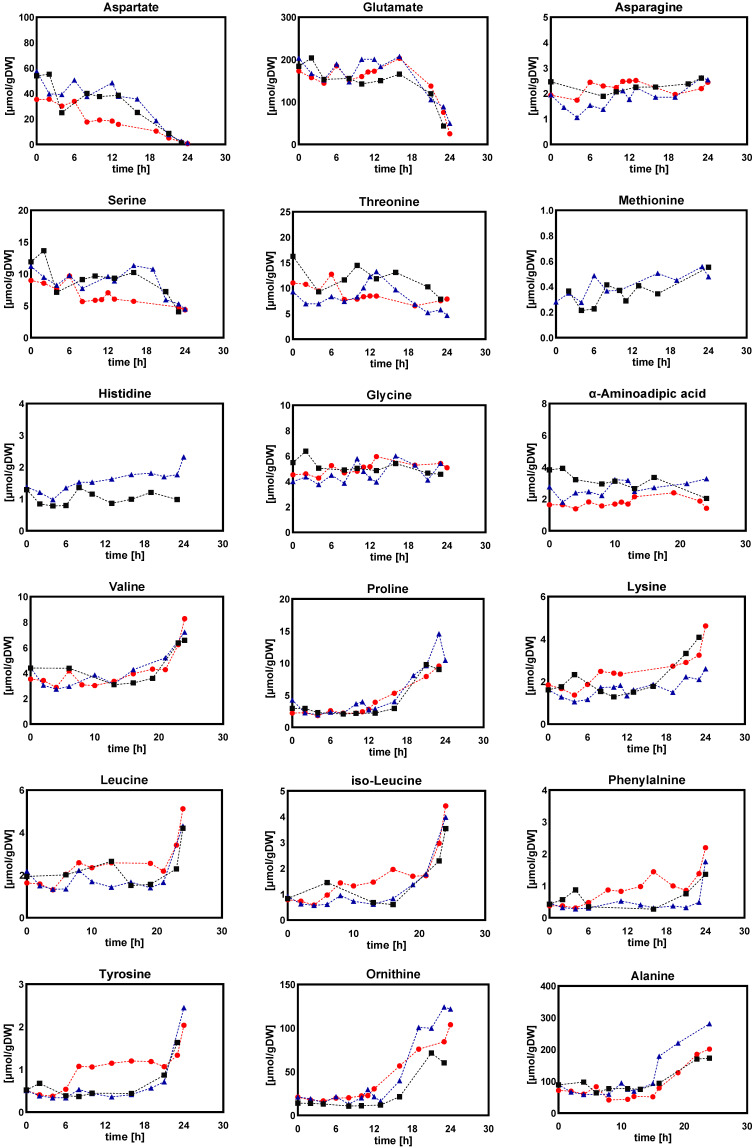
Intracellular amounts of amino acid in *Penicillium chrysogenum* Wisconsin 54-1255 under DO ramp-down experiments. The dissolved oxygen was linearly decreased from 60% to 0% in 24 h. Time 0 represents the start of DO ramp-down. Three legends represent three replicate experiments.

Figure 12 shows the intracellular amounts of amino acids as measured along with a linear decrease of DO level. The results showed that the majority of intracellular amino acids, except for aspartic acid, glutamic acid, serine, and threonine, increased with decreasing DO concentration. However, under DO-shift experiments, some intracellular amino acids were not sensitive to DO perturbations, and their intracellular levels were not significantly altered (Figure 3). Thus, it appeared reasonable that, under the highly dynamic and fluctuating environment of the linear change in DO level, cells did not have enough relaxation time to balance intra- and extracellular metabolism, resulting in large metabolic fluctuations. On the other hand, during steady-state DO-shift conditions, cells gradually adapted to the current DO concentration and thus, their intracellular metabolite concentrations did not greatly fluctuate.

##### Sugar Phosphates, Organic Acids, and Sugar Alcohols

**Figure 13:**
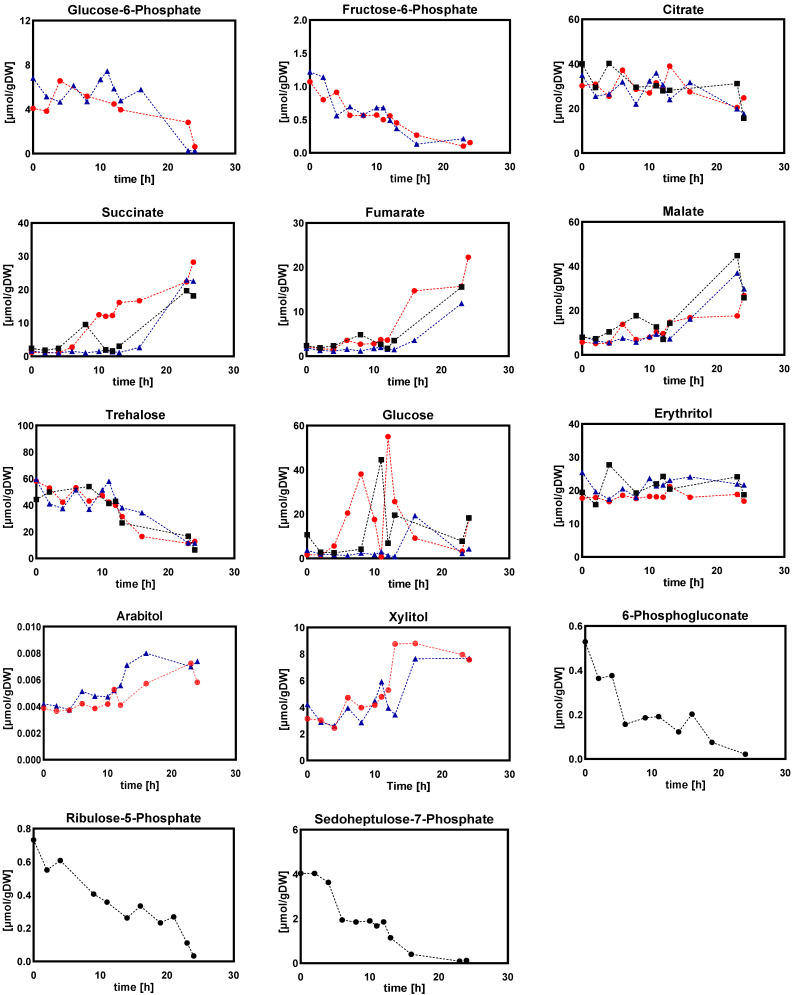
Intracellular amounts of sugar phosphates, organic acids, and sugar alcohols in *Penicillium chrysogenum* Wisconsin 54-1255 under DO ramp-down experiments. The dissolved oxygen was linearly decreased from 60% to 0% in 24 h. Time 0 represents the start of DO ramp-down. Three legends represent three replicate experiments.

Figure 13 shows the dynamics of intracellular amounts of sugar phosphate, organic acid, and sugar alcohol during the DO ramp-down. The results showed that G6P, F6P and S7P decreased under low DO level conditions. This indicated that the metabolism of *Penicillium chrysogenum* shifted under low DO conditions, with a decrease in the amount of glucose entering the PP pathway and an increase in the amount entering the EMP and TCA cycles. Meanwhile, during DO-ramp conditions, the intracellular amounts of malate, succinate, and fumarate involved in the glyoxylate shunt significantly increased, indicating that the glyoxylate shunt was activated under low DO conditions. This was consistent with the results of steady-state DO-shift experiments in this study, collectively demonstrating that *Penicillium chrysogenum* cells were more likely to activate the glyoxylate shunt to reduce the formation of NADH and maintain the redox balance in response to low DO perturbations. In addition, intracellular glucose, arabitol and xylitol were increased under DO ramp-down conditions, which was in agreement with the results of DO-shift experiments. However, intracellular trehalose exhibited different trends under DO shift-down and DO ramp-down experiments, in which it increased and decreased, respectively, implying different regulation mechanisms associated with trehalose formation and transport.

##### Adenine Nucleotides and Energy Charges

**Figure 14:**
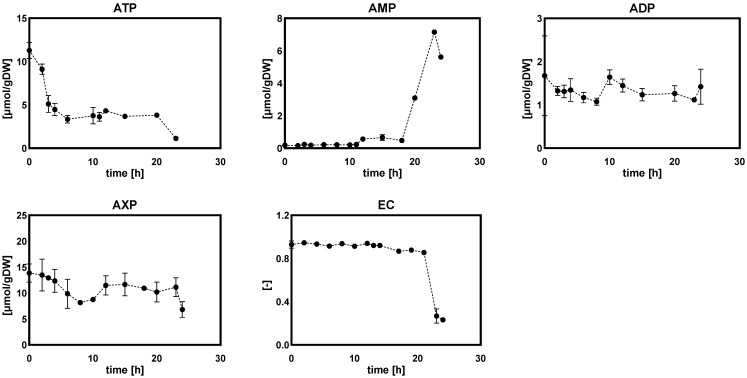
Intracellular amounts of adenine nucleotides and energy charge in *Penicillium chrysogenum* Wisconsin 54-1255 under DO ramp-down experiments. The dissolved oxygen was linearly decreased from 60% to 0% in 24 h. Time 0 represents the start of DO ramp-down.

Figure 14 shows the dynamics of intracellular amounts of adenine nucleotide, as well as energy charge, under DO ramp-down experiments. The intracellular amount of ATP decreased by 60% and leveled off as DO ramped-down to 20%, and continued decreasing when DO reach 0%. ADP did not show significant change, while AMP slowly increased as the DO dropped from 60% to 0% and sharply increased by 90% when the DO was 0%. Interestingly, the total amount of adenine nucleotides remained constant during the DO ramp-down; however, they dropped by 50% when there was a total absence of DO in the broth. In accordance with the change in the intracellular adenine nucleotide level, the energy charge showed robustness to DO perturbation, remained invariable during the DO ramp-down process and, finally, collapsed by 66% when the DO was absent.

##### Cytosolic Redox Status

**Figure 15:**
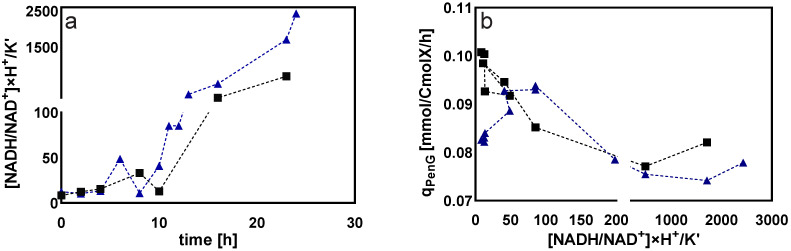
(**a**) Calculated cytoplasmic free NADH/NAD^+^ ratio, assuming a constant intracellular pH and equilibrium constant K′. The dissolved oxygen linearly decreased from 60% to 0% over 24 h. Time 0 represents the start of DO ramp-down. Two legends represent two replicate experiments. (**b**) $q_{\mathrm{PenG}}$ as a function of NADH/NAD^+^ ratio during the DO ramp-down experiments.

In this study, the C_4_ equilibrium pool (aspartate and malate) in the tricarboxylic acid cycle was used to estimate the cytosolic redox status in the form of NADH/NAD^+^ ratio [39]. As shown in Figure 15a, the cytoplasmic free NADH/NAD^+^ ratio was gradually increased before 10 h (DO at 10%), followed by a sharp increase during the DO ramp-down experiment.

As shown in Figure 15b, the $q_{\mathrm{PenG}}$ decreased with an increasing cytoplasmic reduction state. A high cytoplasmic reduction state may have affected the activity of the relevant enzymes in penicillin synthesis and thus reduced its production. Consistently, our previous studies have also concluded that there could exist an inverse relation between the cytosolic NADH/NAD^+^ ratio and penicillin production capacity [29,39].

##### Extracellular Sugar Phosphates, Organic Acids, and Sugar Alcohols

**Figure 16:**
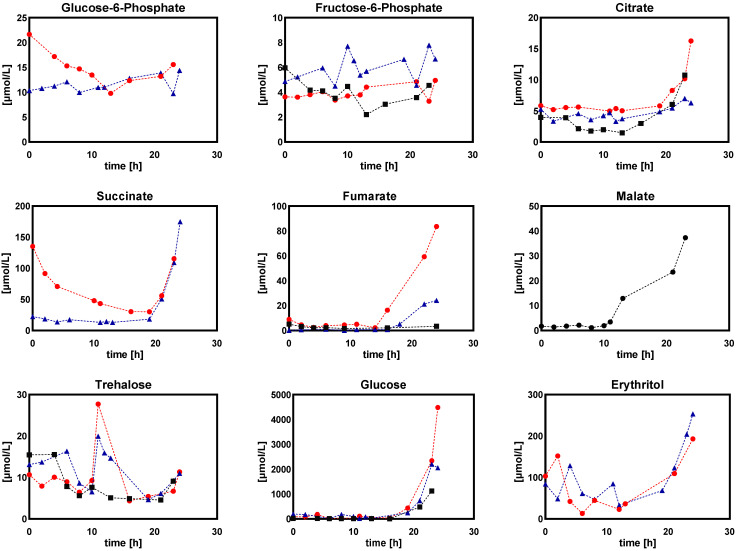
Extracellular concentrations of sugar phosphates, organic acids, and sugar alcohols in *Penicillium chrysogenum* Wisconsin 54-1255 under DO ramp-down experiments. The DO linearly decreases from 60% to 0% over 24 h. Time 0 represents the start of DO ramp-down. Different legends represent replicate experiments.

Figure 16 shows the dynamics of extracellular concentrations of sugar phosphate, organic acid, and sugar alcohol under DO ramp-down conditions. Consistent with the results of the DO shift-down experiments in this study, collectively, organic acids (except succinate) showed an increasing trend while sugar phosphates showed a decreasing trend as the DO dropped down (Figure 7). Strikingly, the extracellular glucose concentration showed an approximately threefold increase (14 µM) after the DO was shifted down to 5%, while the extracellular glucose concentration increased to about 100 µM when the DO was ramped down to 5%. This indicated that *Penicillium chrysogenum* cells were robust to long-term perturbation of low DO levels under DO-shift conditions, and became sensitive to short-term perturbation of low DO levels under DO-ramp conditions.

##### Ratio of Extra/Intracellular Metabolite Concentrations

**Figure 17:**
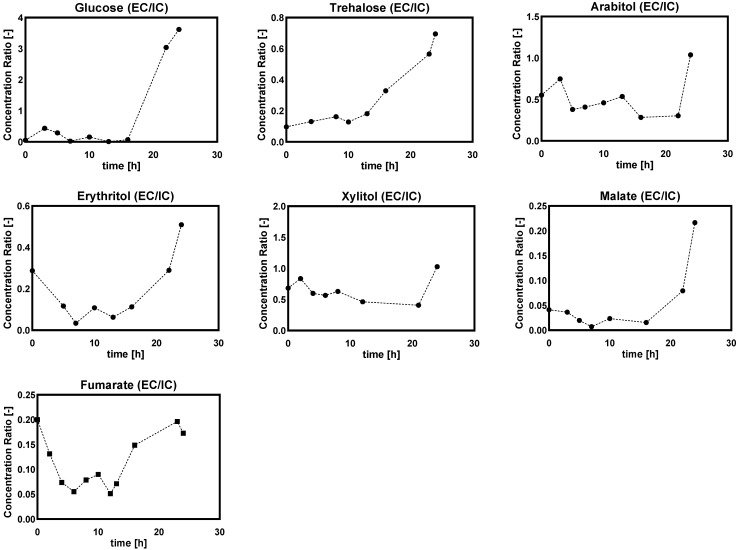
The ratio of extra/intracellular (EC/IC) metabolite concentrations (assuming a cellular volume of 2.5 mL/gDW) in *Penicillium chrysogenum* Wisconsin 54-1255 under DO ramp-down experiments. The dissolved oxygen was linearly decreased from 60% to 0% in 24 h. Time 0 represents the start of DO ramp-down.

Figure 17 shows the ratio of EC/IC concentration of sugar alcohols during the DO ramp-down condition. All measured extracellular metabolites were kept constant before 10 h (DO at 10%) after the start-up of the DO ramp-down, followed by a linear increase with the decrease of the DO level. The higher EC/IC ratio implied that these measured metabolites had increased export or leakage during the DO ramp-down process. However, compared with the results of the DO shift-down experiments, glucose EC/IC ratio showed the same trend in both DO shift and DO ramp-down, while trehalose ratios differed between the two experiments (Figure 8 and Figure 17). Additionally, we did not observe significant change in the EC/IC ratio of other metabolites in this *Penicillium chrysogenum* strain during the DO shift-down experiment, which indicated that different timescales of DO perturbation gave rise to utterly different metabolic responses.

## 3. Discussion

Apart from glucose as the source of energy and carbon in penicillin production by *Penicillium chrysogenum*, oxygen is used both as the final electron acceptor for ATP production and as the direct reactant in the second step of penicillin biosynthesis. Therefore, it is of vital importance to gain quantitative insight into the cellular regulation mechanism in response to relevant DO perturbations typically occurring in large-scale settings. In this study, the DO-shift and DO-ramp strategies were used to generate a wide range of DO levels experienced by an industrial strain of *Penicillium chrysogenum*, Wisconsin 54-1255. The results showed that penicillin productivity was decreased with decreasing DO concentration, and that lower DO concentration corresponded to lower penicillin production capacity. However, in comparison with the reference control at the DO level of 60%, the $q_{\mathrm{PenG}}$, under 5% steady-state dissolved oxygen conditions, decreased by 25%, while decreasing by only 14% when the DO was linearly reduced from 60% to 0%. This implied that prolonged exposure to a low DO environment was the detrimental factor leading to decreased penicillin production capacity.

From the results of DO-shift and DO-ramp experiments, we deduced three possible reasons for the decrease in penicillin production by *Penicillium chrysogenum* in response to the low dissolved oxygen perturbation: (a) inadequate energy supply. In their study, van Gulik et al. [28] performed an in-depth analysis of the energy metabolism in the biosynthesis of penicillin by a high-yielding *Penicillium chrysogenum* strain using extensive chemostat experimental data. They estimated the energy parameters used for growth, penicillin synthesis and maintenance. It was estimated that 73 mol ATP were required for the production of 1 mol penicillin [28], and further study showed that this large requirement for ATP was mainly ascribed to the futile cycling of ATP-free PAA passive uptake and ATP-consuming PAA active export for detoxification [40]. It was evident that the intracellular ATP decreased significantly in response to both DO shift-down and DO ramp-down conditions (Figure 6 and Figure 14), and that this insufficient ATP supply would most likely lead to a decrease in penicillin productivity. (b) Unbalanced redox status could affect the function of PenG biosynthetic enzymes. The cytosolic redox status, which was indicated by NADH/NAD^+^ using the metabolite sensor reaction, showed an increasing trend as the DO decreased (Figure 15a). Meanwhile, the $q_{\mathrm{PenG}}$ decreased with increases in the cytoplasmic reduced state (Figure 15b). A possible explanation is that a more reduced cytosol affected the reduction of disulfide bonds in the relevant enzymes, which in turn affected the folding and function of the protein as well as its binding properties and gene expression [41]. Previous studies have also reported that expression of impaired or unbalanced penicillin synthesis enzymes such as L-α-(δ-aminoadipyl)-L-α-cy-steinyl-D-α-valine synthetase (ACVS), isopenicillin-N synthetase (IPNS) and isopenicillin N acyltransferase (IAT) in the high-yielding *Penicillium chrysogenum* can lead to a rapid reduction in penicillin biosynthesis capacity [38,42]. (c) Increase in extracellular glucose concentration can cause glucose suppression [43,44,45,46,47,48]. For example, Revilla et al. found that the first intermediate of the penicillin biosynthetic pathway, α-aminoadipoyl-cysteamine-valine, was reduced when *Penicillium chrysogenum* were grown in high concentrations of glucose [47]. This is because glucose stimulates flux through the lysine biosynthetic pathway, thereby preventing the accumulation of aminoadipic acid. Additionally, glucose inhibits isopenicillin N synthase, the last enzyme in the penicillin synthesis pathway [47]. Obviously, in this study, the impaired respiration metabolism gave rise to increased extracellular glucose concentrations under the low DO levels (Figure 7 and Figure 16). The extracellular glucose concentrations in 5% DO were three times and 25 times higher, respectively, than in 60% DO conditions under the DO shift and ramp conditions. Hence, the high glucose environment under low DO conditions could lead to decreases in the activity of the enzyme associated with penicillin biosynthesis.

Furthermore, quantitative metabolomics, in combination with fluxomics, was used to gain a quantitative view of intracellular metabolic flux distribution under these DO-perturbation experimental conditions. The results showed that intracellular metabolites such as sugar phosphate and sugar polyol were inclined to be accumulated for carbon storage as well as redox balance in response to the low DO conditions (Figure 5 and Figure 13). Meanwhile, it was interesting to observe that the transport of sugar polyols changed, showing an active exportation trend when the DO was gradually decreased (Figure 17). Consistent with the impaired energy metabolism, the total adenine nucleotide (AXP) and energy charge were significantly decreased when the DO was lower than 10%. Moreover, the majority of intracellular metabolites went up during the DO ramp-down, while amino acids were less sensitive during the DO shift. It is possible that the highly dynamic and fluctuating environment, with linear changes in the DO level, in which cells lacked sufficient relaxation time to balance intra- and extracellular metabolism, led to more pronounced metabolic responses. Remarkably, the glyoxylate shunt was very likely to be enforced during the low DO perturbation. This manifested in the significant increase of intra- and extracellular malate, succinate, and fumarate involved in the TCA cycle (Figure 5 and Figure 13). The enhanced glyoxylate shunt was reported as an important cellular regulation strategy to reduce intracellular NADH production and maintain intracellular homeostasis [20]. In addition, this was further demonstrated by the increased flux of the glyoxylate branch using flux balance analysis (Figure 9).

In the present study, all of these DO-ramp and DO-shift experiments were performed to provide informative insights into the metabolic responses of *Penicillium chrysogenum* to DO perturbation. However, these DO perturbation experiments were carried out over a matter of hours, a timescale that is not highly relevant for industrial-scale practices wherein microorganisms primarily experience DO dynamics at timescales of tens of seconds or minutes [49]. Therefore, it must be noted that the relevant DO perturbation-based metabolic response and cellular regulation strategy in this industrially relevant *Penicillium chrysogenum* should be further investigated in down-scaling experiments that account for microorganism’s lifelines in industrial-scale bioreactors.

## 4. Materials and Methods

### 4.1. Materials

The *Penicillium chrysogenum* Wisconsin 54-1255 was purchased from ATCC, fungal spores were prepared on potato dextrose agar medium (PDA), and the concentrated spore suspensions were stored uniformly in aqueous glycerol solution (30% (*v*/*v*) glycerol in demineralized water) at −80 °C. Spore suspension inoculation was used in all experiments and were prepared to ensure that the final spore concentration in the bioreactor after inoculation was about 1 × 10^6^/mL, as described previously [29].

### 4.2. Fermentation Media

The composition of the medium for the batch phase and chemostat cultivation was the same as previously described, containing (g/kg): 16.5 g C_6_H_12_O_6_·H_2_O, 5 g (NH_4_)_2_SO_4_, 1 g KH_2_PO_4_, 0.5 gMgSO_4_·7H_2_O, 2 mL trace elements, 1 mL antifoaming agent. The trace element composition (per kg of deionized water) was 75 g Na_2_EDTA·2H_2_O, 10 g ZnSO_4_·7H_2_O, 10 g MnSO_4_·1H_2_O, 20 g FeSO_4_·7H_2_O, 2.5 g CaCl_2_·2H_2_O, 2.5 g CuSO_4_·5H_2_O. The phenylacetic acid (PAA) concentrations in the batch and the chemostat media were supplied at 0.4085 g/kg and 0.68 g/kg, respectively, which was neither limiting nor toxic to cell growth throughout the cultivation [40]. The preparation and sterilization of the cultivation medium have been described previously [50]. Briefly, glucose solution and the PAA-containing salt solution were prepared and sterilized at 110 °C and 121 °C for 40 min and 30 min, respectively. The PAA was dissolved in a KOH solution, with a PAA: KOH molar ratio of 1:1.2 [51].

### 4.3. Chemostat Cultivation

Glucose-limited chemostat conditions with a working volume of 3 L were carried out in a 5 L turbine-stirred bioreactor (Shanghai Guoqiang Bioengineering Equipment Co., Ltd., Shanghai, China) at a dilution rate of 0.05 $h^{-1}$. When the glucose in the batch phase was depleted, the culture was switched to chemostat cultivation. The broth was drained by a peristaltic pump through an overflow tube located at the bioreactor gas/liquid interface. The dissolved oxygen tension (DOT) was detected with an oxygen probe (Visiferm DO HAMILTON, Switzerland), which never dropped below 60% of air saturation (calibrated at 100% at 0.4 bar overpressure). The temperature was maintained at 25 °C, the airflow rate was set at 2 L/min, the stirrer speed was set at 600 RPM and pH was controlled at 6.5 with 4 M sodium hydroxide using a sterilizable pH probe (Mettler–Toledo, Greifensee, Switzerland). The off-gas oxygen and carbon dioxide fractions were monitored in real-time using an off-gas mass spectrometer (Prima BT, Thermo Fisher Scientific Winsford U.K.CW7 3GA) [39].

(a) DO shift-down experiment

Four DO shift-down experiments were performed, during which the DO concentration was shifted from 60% down to 20%, 10% and 5%, in turn. The chemostat cultivation at the four dissolved oxygen concentrations lasted for 200 h. During the DO shift-down phase, changes in the ratio of nitrogen to compressed air were used to modulate the DO level, while the total air flow rate remained unchanged at 2 L/min.

(b) DO ramp-down experiment

DO ramp-down experiments were performed in which the DO was linearly decreased from 60% to 0% over 24 h. The ramp-down experiment began after the chemostat system initially reached steady-state at a DO level of 60%. During the ramp phase, the fermentation settings remained the same as during the chemostat phase, except the airflow rate was linearly decreased from 2.0 to 0 L/min to facilitate a linear decrease in the dissolved oxygen concentration.

**Figure 18:**
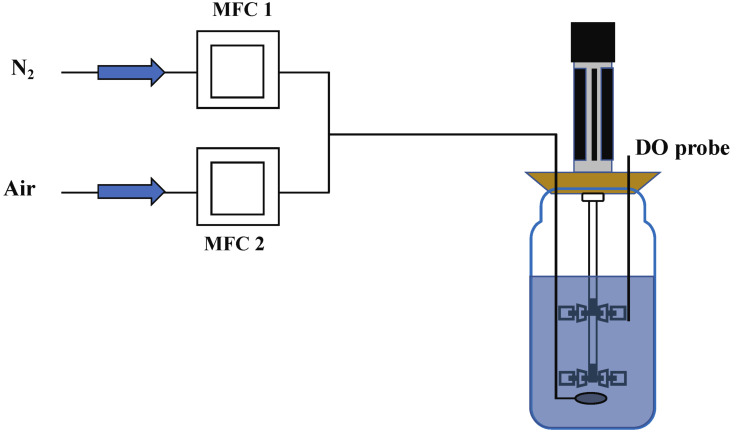
A schematic overview of nitrogen and compressed air control logic used in the DO shift and ramp experiments.

Gas proportional regulation for DO ramp and shift was achieved using two mass flow controllers (MFC). The design of the mass flow controller for chemostat systems is shown in Figure 18. In this case, MFC 1 was responsible for regulating the absolute flux of nitrogen in the inlet gas, while MFC 2 was responsible for the absolute flux of air in the inlet gas. The nitrogen and air were mixed and passed into the reactor to modulate the dissolved oxygen level.

### 4.4. Cell Dry Weight

Cell dry weight (CDW) was measured by weight difference between empty glass fiber filters and dry glass fiber filters (47 mm in diameter, 1-µm pore size, type A/E; Pall Corporation, East Hills, NY, USA) with biomass. About 15 mL of broth was extracted and divided into three portions for CDW measurements [29]. For each CDW sample, 5 mL of the broth was filtered, and the cell cake was washed three times with 10 mL of demineralized water and dried in a microwave oven (power 800 W) for 4 min. The filter containing biomass was cooled to room temperature in a desiccator and weighed.

### 4.5. Rapid Sampling, Quenching and Metabolite Extraction

Under DO-shift conditions, samples were taken at each residence time. Under DO-ramp conditions, samples were taken at every 10% decrease in dissolved oxygen (60%, 50%, 40%, 30%, 20% and 10%) until the dissolved oxygen fell below 10%. Then, the sampling frequency was increased to coincide with every 1% decrease in the DO level (5%, 3%, 2%, 1% and 0%) for the determination of both intra- and extracellular metabolites.

For extracellular sampling, the cold steel-bead method, combined with liquid nitrogen, was used for the fast filtration and quenching of extracellular enzyme activities, as described previously [52]. For intracellular sampling, approximately 1.2 mL of broth was taken from the bioreactor into a tube containing the quenching solution (−27.5 °C, 40% *v*/*v* aqueous methanol). A customized rapid sampling device was developed to ensure fast sampling and rapid quenching, avoiding, insofar as possible, changes in intracellular metabolites. For detailed sampling procedures, please refer to Li et al. [53]. Rapid filtration and an improved cold wash method were used to quickly and efficiently remove all compounds present outside the cells. In this study, a previously established protocol for rapid sampling, quenching and subsequent metabolite extraction was followed, as previously described [54]. For quantification of intracellular metabolites, isotope dilution mass spectrometry (IDMS) was used to correct most aspects of analytical bias [55]. U-^13^C-labeled cell extracts obtained from *Saccharomyces cerevisiae*-supplemented batch cultures were added as internal standards in MS-based metabolite analysis [56].

### 4.6. Analytical Methods

Quantification of intracellular amino acids, sugar phosphates, organic acids, and sugar alcohols was performed by gas chromatography-mass spectrometry (GC-MS) (7890A GC coupled to 5975C MSD; Agilent Technologies, Santa Clara, CA, USA). The analytical procedures were performed according to de Jonge et al. [57], with slight modifications in the column and temperature gradients. For specific settings, please refer to Liu et al. [58].

An Agilent Zorbax SB-C_18_ reversed-phase column (150 mm × 4.6 mm ID, 5 µm) was used to determine the concentrations of PAA, PenG, and byproducts in the penicillin biosynthetic pathway using equal gradient, reversed-phase high-performance liquid chromatography (HPLC). The mobile phase was 0.44 g/L potassium dihydrogen phosphate in acetonitrile/water (65/35, *v*/*v*); the injection volume was 5 µL, the detection wavelength was 214 nm, the flow rate was 1.5 mL/min and the column temperature was 25 °C [52].

As for metabolomics data, the principal component analysis (PCA) and partial least square discriminant analysis (PLS-DA) were conducted based on the R programming language. If the variable importance of the projection (VIP) score of one metabolite was above 1, the pool size of this metabolite changed significantly [20].

### 4.7. Adenylate Energy Charge

The energy charge (EC) was calculated as follows [59]:

$$\mathrm{EC}=\frac{\mathrm{ATP}+\left(\frac{\mathrm{ADP}}{2}\right)}{\mathrm{ATP}+\mathrm{ADP}+\mathrm{AMP}}$$

### 4.8. Cytosolic Free NADH/NAD^+^ Ratio

The cytosolic free NADH/NAD^+^ ratio was calculated using an equilibrium pool of C_4_ (aspartate and malate) in the TCA cycle under the assumption of constant K′ and intracellular pH [60]:

$$\frac{\mathrm{NADH}}{{\mathrm{NAD}}^{+}}=K^{\prime }\frac{\mathrm{Malate}*\mathrm{Glutamate}}{{\alpha \mathrm{KG}*\mathrm{Aspartate}*H}^{+}}$$

### 4.9. Genome-Scale Metabolic Network Model

The genome-scale metabolic model (GEM) of *Penicillium chrysogenum* Wisconsin 54-1255, consisting of 1471 unique biochemical reactions and 1006 ORFs, was used to estimate the metabolic flux distribution under the DO-perturbation experiments [36].

## Figures and Tables

**Table 1:** Comparison of the biomass-specific rates and relevant yields of *Penicillium chrysogenum* Wisconsin 54-1255 on glucose obtained from DO-shift cultures at the average dilution rate of 0.05 $h^{-1}$ in the time range of 100–300 h of cultivation. Measurements are given as average ± standard deviation of at least two individual experiments.

		60%	20%	10%	5%
−$q_{s}$	mmol/CmolX/h	18.06 ± 1.89	16.10 ± 0.95	16.05 ± 0.56	14.88 ± 0.76
−$q_{O_{2}}$	mmol/CmolX/h	73.09 ± 9.44	68.84 ± 1.1	70.52 ± 4.55	61.65 ± 4.24
$q_{{\mathrm{CO}}_{2}}$	mmol/CmolX/h	77.16 ± 10.3	72.68 ± 1.1	70.49± 3.80	61.73 ± 3.84
−$q_{\mathrm{PAA}}$	mmol/CmolX/h	0.23 ± 0.03	0.22 ± 0.04	0.22 ± 0.06	0.19 ± 0.02
$q_{\mathrm{PenG}}$	mmol/CmolX/h	0.089 ± 0.002	0.080 ± 0.004	0.076 ± 0.004	0.067 ± 0.004
$q_{\mathrm{OPC}}$	mmol/CmolX/h	0.26 ± 0.04	0.41 ± 0.03	0.51 ± 0.04	0.44 ± 0.07
$q_{6\mathrm{APA}}$	mmol/CmolX/h	0.42 ± 0.07	0.39 ± 0.05	0.36 ± 0.04	0.33 ± 0.04
$q_{o-\mathrm{OH}-\mathrm{PAA}}$	mmol/CmolX/h	0.15 ± 0.03	0.20 ± 0.13	0.20 ± 0.044	0.12± 0.01
$C_{X}$	g/kg	6.68 ± 0.64	7.36 ± 0.51	7.36 ± 0.30	8.19 ± 0.59
$Y_{X/S}$	g/g	0.45 ± 0.04	0.49 ± 0.02	0.50 ± 0.03	0.54 ± 0.03
$Y_{O/S}$	mol/mol	4.46 ± 0.86	4.34 ± 0.10	4.40 ± 0.01	4.26 ± 0.44
$Y_{P/S}$	mmol/mol	4.95 ± 0.66	5.07 ± 0.32	4.99 ± 0.17	4.99 ± 0.01
RQ	-	1.06 ± 0.01	1.05 ± 0.01	1.07 ± 0.07	1.08 ± 0.01

## Data Availability

The data presented in this study are available on request from the corresponding author because of its usage in the ongoing study.
